# The green code of heating revolution: exploring the impact of China's northern clean heating pilot on the green total factor energy efficiency

**DOI:** 10.3389/fpubh.2026.1854174

**Published:** 2026-05-29

**Authors:** Lanyue Zhang, Tingting Long, Na Yu

**Affiliations:** 1 School of Digital Economics, Sichuan University Jinjiang College, Meishan, Sichuan, China; 2 College of Management Science, Chengdu University of Technology, Chengdu, Sichuan, China; 3 College of Humanities and Law, Chengdu University of Technology, Chengdu, Sichuan, China; 4 School of Economics and Management, Inner Mongolia University, Hohhot, Inner Mongolia, China

**Keywords:** clean heating policy, green total factor energy efficiency, influence mechanism, multi-period difference-in-differences model, porter hypothesis

## Abstract

To address severe air pollution caused by winter heating, China has implemented a mandatory clean heating policy in northern regions. Although the environmental benefits of this policy have been recognized, its net impact on green total factor energy efficiency (GTFEE) and the underlying mechanisms remain unclear. This paper regards this policy as a quasi-natural experiment. Based on the panel data of 300 Chinese cities from 2006 to 2022, we use the multi-period difference-in-differences model for causal identification. We find that the GTFEE of pilot cities increased by an average of about 6.5% after policy implementation, and this finding remains robust after excluding contemporaneous environmental policies, providing empirical evidence from China for the Porter hypothesis. Heterogeneity analysis shows that policy effects are more prominent in non-coastal cities, high-tech endowed cities, and high administrative level cities, reflecting the key role of local capacity in policy implementation. The mechanism inspection identified four main transmission paths: industrial structure upgrading, government governance capacity improvement, environmental regulation strengthening, and green technology progress. This study not only provides new evidence from northern China for the Porter effect induced by command-based environmental regulations, but also suggests that future environmental policies should focus more on matching local endowments to achieve precise governance and synergistic efficiency.

## 1 Introduction

In the grand context of global energy transition and regional environmental governance, balancing economic growth and environmental protection has become a core challenge faced by countries around the world, especially rapidly developing countries (1). This challenge is closely related to the goals of “Ensuring affordable, reliable, and sustainable modern energy for all” (SDG 7) and “Building inclusive, safe, resilient, and sustainable cities and human settlements” (SDG 11) in the United Nations' 2030 Agenda for Sustainable Development (2). Due to its unique climate conditions and energy structure, scattered coal combustion during the winter heating season in northern China has long been an important contributor to regional haze, causing enormous environmental and health pressures (3, 4). To address this serious issue, the Chinese government has systematically implemented clean heating policies in northern regions since 2017, aiming to reduce civilian coal consumption from the source through projects such as “coal to gas” and “coal to electricity”. This policy practice also provides an important research opportunity for exploring how to coordinate energy sustainability and urban environmental governance.

Existing research on clean heating policies mostly focuses on their direct impact on air quality improvement, residents' health benefits, and energy consumption (5), providing valuable insights into understanding the end environmental effects of policies. For air quality, the policy has been found to significantly reduce SO_2_ by 20.4% through energy structure optimization, but does not significantly reduce PM_2.5_ (58). Another study reported a 12.3-unit decrease in the Air Quality Index and a reduction in lung disease among middle-aged and middle-aged residents, though household heating costs increased (6). In terms of health and economic benefits, the policy lowered PM_2.5_ by 3.7%−31.7%, reduced PM_2.5_-related health outcomes by 7.7%−52.9%, and averted 3.197 billion yuan in economic losses (7). At the household level, clean heating adoption is driven by income, education, subsidies, and awareness of indoor air pollution, with notable energy stacking behavior (8). Regarding carbon emissions, the policy reduced unit GDP and per capita carbon intensity but had no significant effect on total emissions; it exhibited a multiplier effect and structural effect but no Porter effect, with emissions even increasing in coal power-exporting regions (9). However, a perspective worthy of further exploration is whether and to what extent the energy substitution and technological innovation driven by this policy have promoted deep optimization of the economic energy environment system, i.e., improved green total factor energy efficiency (GTFEE) (10). A thorough analysis of this issue can help evaluate more comprehensively the comprehensive benefits of clean heating policies (11).

In addition to command-and-control environmental regulations, existing research has begun to focus on the driving effects of various emerging factors on green total factor energy efficiency. For example, digital technology innovation (12) and digital finance have improved energy efficiency by optimizing resource allocation; Green fiscal policies (13) have been proven to effectively narrow the energy efficiency gap; In addition, the promotion of new energy vehicles (14) and the construction of urban rail transit (15) have also been found to be important paths to improve urban GTFEE. Beyond these factors, industrial robot adoption has been found to promote GTFEE by facilitating industrial agglomeration and enhancing technological innovation capacity, particularly green technology innovation; this effect is stronger in resource cities from the perspective of the “resource curse” (16). Climate policy uncertainty can also improve urban GTFEE, operating through heightened public environmental concerns and the optimization of energy consumption structures, with more pronounced effects in resource-based and economically developed cities (17). At the firm level, green mergers and acquisitions lead to a roughly 1%−2% increase in GTFEE among heavy polluting enterprises, a gain that is mediated by stronger green technology innovation and greater access to government subsidies, and that is more evident in labor-intensive industries and less competitive markets (18). Meanwhile, the digital economy has been shown to enhance corporate resilience in part by fostering green innovation, a relationship in which green total factor energy efficiency serves as a positive moderator, particularly in state-owned enterprises (19). At the sectoral level, GTFEE across China' s industrial sub-sectors has exhibited a rising trend driven largely by technological progress, though substantial heterogeneity exists; high-energy-consumption sub-sectors display only conditional beta convergence, whereas low-to-medium-energy-consumption sub-sectors show both sigma and beta convergence (20). These studies collectively suggest that the clean heating policy may affect GTFEE through similar mechanisms involving structural change and technological innovation.

In terms of research methods, although the difference in differences model has become a commonly used method for policy evaluation, it is still necessary to maintain rigor in research design when applied to such multi period policies (21). Compared with the conventional two-period DID design, the multi-period framework exploits richer temporal and cross-sectional variation arising from the gradual rollout of policies, thereby accommodating dynamic treatment effects and circumventing the confounding influence of contemporaneous shocks that often undermine single-event studies. Moreover, relative to alternative causal inference approaches, such as synthetic control methods that are typically restricted to one or a few treated units, or regression discontinuity designs that rely on arbitrary cutoff points rarely available in nationwide clean heating programs, the multi-period DID model offers a flexible, transparent, and widely applicable identification strategy for evaluating regionally batch-implemented command-and-control regulations. Nevertheless, ensuring the reliability of such estimates still necessitates careful testing of parallel trends, handling of potential endogeneity, and systematic analysis of heterogeneity and influencing mechanisms. Especially, as a typical command and control environmental regulation, the effectiveness of clean heating policies may be influenced by initial conditions and resource endowments in different regions (22). For example, the path dependence formed by resource-based cities' long-term dependence on traditional energy and the transformation potential possessed by cities with higher technological endowments may result in different implementation effects of the same policy (23). Therefore, in-depth analysis of the differences in policy effectiveness among different types of cities has reference value for optimizing subsequent policy design.

**Figure 1:**
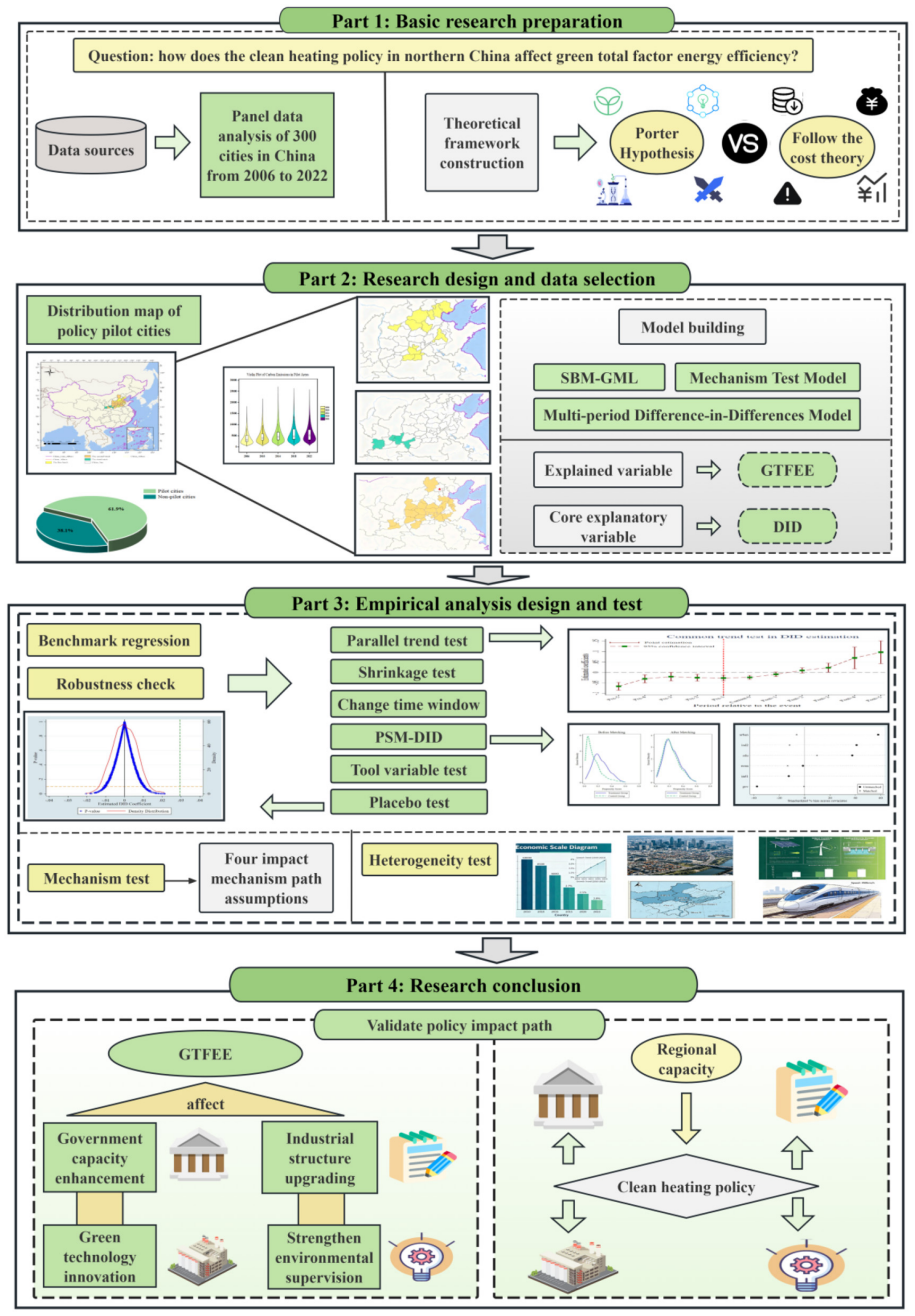
Research framework.

In this context, our study aims to systematically assess the impact of the northern clean heating policy on green total factor energy efficiency (GTFEE) through a rigorous causal inference framework (as shown in Figure 1). The innovative contributions of this paper are mainly reflected in the following three aspects. First, we shift the evaluation paradigm from terminal environmental outcomes to systemic energy, economy, and environment efficiency. By providing causal evidence that a command-and-control clean heating policy significantly improves green total factor energy efficiency, we offer novel urban-level support for the Porter hypothesis in a mandatory regulation context where such evidence is scarce. Second, we develop and empirically substantiate the concept of “regional absorptive capacity.” We demonstrate that the policy-induced efficiency gains are systematically larger in non-coastal, high-tech endowed, and high-administrative-level cities, revealing that the effectiveness of uniform environmental mandates is endogenously conditioned by local structural and institutional capabilities, an insight that directly informs precision-tailored governance. Third, we simultaneously identify four synergistic transmission channels, namely industrial structure upgrading, governance capacity enhancement, environmental regulation tightening, and green technology progress, and establish green innovation as the core direct driver. This multi-path mechanism provides a micro-foundational account of how mandatory environmental policies deliver the dual dividends of pollution reduction and efficiency improvement.

## 2 Institutional background, theoretical analysis and research hypothesis

### 2.1 Background of northern clean heating policy

The northern clean heating policy is a key measure implemented by China to control air pollution and promote energy transformation. The policy took the lead in launching a pilot in Beijing Tianjin Hebei and surrounding “2 + 26” cities in 2017, with the core goal of solving the serious pollution problem caused by the combustion of loose coal in winter heating season (11). In the initial stage, the “coal to gas” and “coal to electricity” were the main technical paths, relying on the central and local financial subsidies. In 2018, the scope of the policy was extended to key areas such as the Fenwei plain, and the technical route became more diversified. The principle of “electricity is appropriate, gas is appropriate, and coal is appropriate” was emphasized, and the application of renewable energy in the field of heating was explored (15).

**Figure 2:**
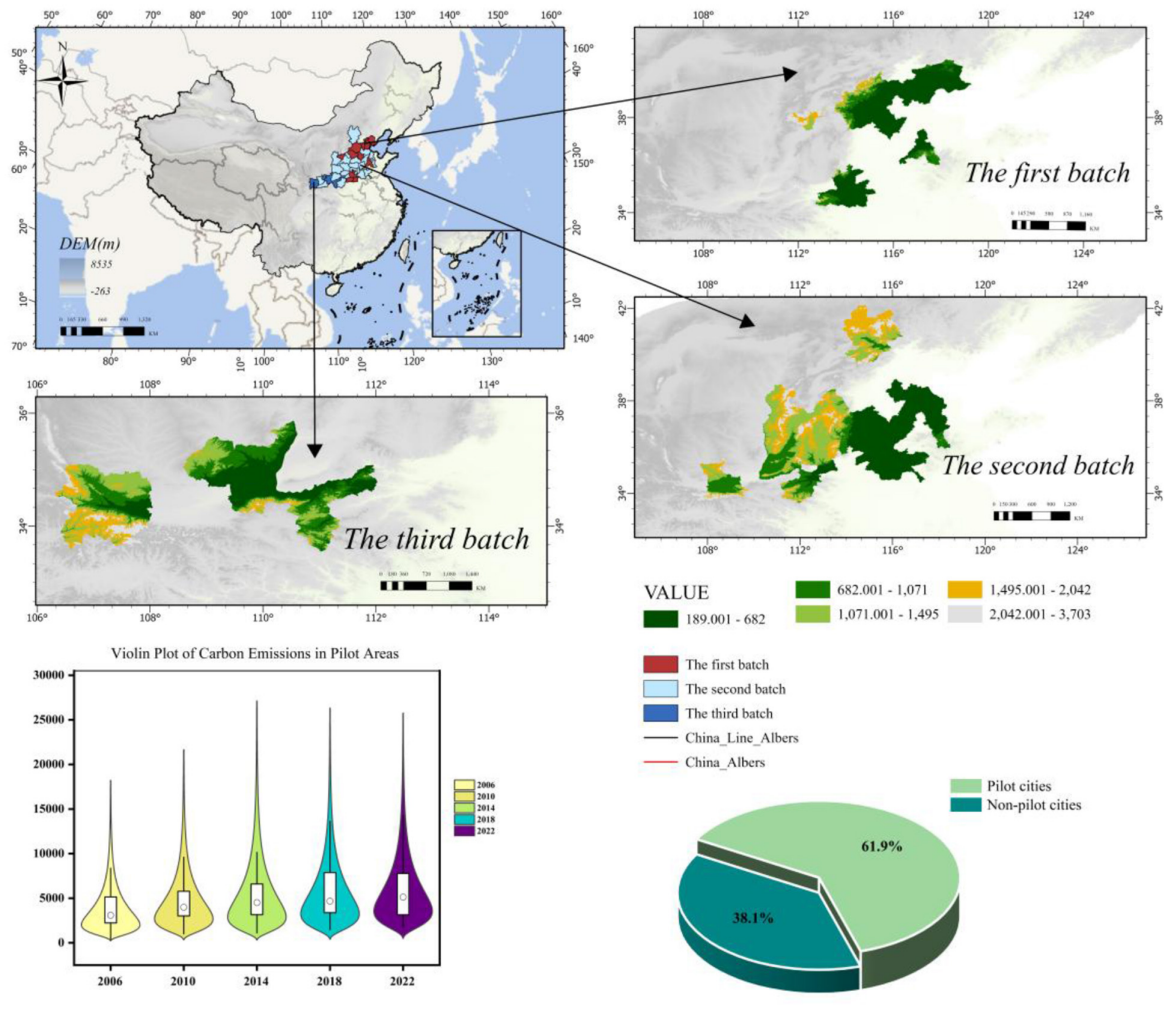
Different batches of pilot areas.

Since 2019, the policy has entered the stage of deepening and improving, and its focus has shifted from the initial scale expansion to the establishment of a long-term mechanism and the improvement of quality and efficiency. Policy pilot city changes are shown in Figure 2. The guidance jointly issued by the national development and Reform Commission, the energy administration and other departments marks this strategic change. At this stage, efforts will be made to improve the electricity and gas price mechanism, strengthen equipment operation and maintenance and energy supply guarantee, so as to consolidate the existing transformation achievements and prevent the risk of “coal return”. The promotion of the policy in the pilot cities has formed a typical “quasi natural experiment” based on their air quality improvement needs and resource endowment conditions, which provides a solid research foundation for the systematic evaluation of their environmental, economic and social benefits (14, 23).

### 2.2 Theoretical analysis

The impact mechanism of environmental regulation on regional energy efficiency is the focus of this study. The existing research on environmental regulation has been debating around the “Porter Hypothesis” and the “Compliance Cost Theory” for a long time (24, 25). The former believes that properly designed environmental regulations can stimulate innovation compensation, while the latter emphasizes the compliance cost and technical rigidity it brings. As for China' s clean heating policy, most of the existing literatures focus on its direct impact on air quality improvement, residents' health benefits and energy consumption. These studies provide valuable insights for us to understand the end environmental effects of the policy (26). However, a perspective worth in-depth discussion is that energy substitution and technological innovation driven by policies may trigger broader efficiency changes in the entire economic system, and GTFEE is the core indicator to capture this synergy process.

As a typical representative of command-and-control environmental regulation, clean heating policy may affect green total factor energy efficiency through multiple paths. Our research starts from the following four paths.

#### 2.2.1 Industrial structure transformation

The clean heating policy promotes the optimization and upgrading of regional industrial structure by changing the energy consumption structure. The implementation of the policy directly reduced the demand for the traditional coal industry chain, and promoted the development of clean energy equipment manufacturing, energy-saving technology services and other related industries (59). This structural change promotes the transfer of production factors from high energy consumption and high pollution sectors to clean and efficient sectors, thereby improving the efficiency of regional energy resource allocation (27). Existing studies have shown that the adjustment of industrial structure caused by environmental regulation is an important way to improve regional green total factor productivity (28). The study further points out that resource efficiency actions, such as energy conservation and the use of renewable energy, have a positive impact on economic and environmental performance, but their effect is regulated by production costs, highlighting the importance of cost-benefit balance in the transformation process (29, 30).

#### 2.2.2 Strengthening government governance

The implementation process of clean heating policy is also the process of improving the environmental governance ability of local governments. The policy incorporates the goal of clean heating into the local assessment system, urges local governments to improve the environmental supervision mechanism, optimize the allocation of financial funds, and strengthen departmental coordination and linkage. This improvement of governance capacity will help to improve the efficiency of policy implementation and ensure the stable operation and sustainable utilization of clean energy facilities. Research shows that the enhancement of local governments' environmental governance capacity plays an important role in improving the effect of policy implementation (31).

#### 2.2.3 Strengthening environmental regulation

The mandatory characteristics of the clean heating policy have sent a clear policy signal to the market players and changed the environmental compliance expectations of enterprises (32). In order to meet increasingly stringent environmental protection requirements, enterprises need to increase investment in energy-saving and environmental protection equipment and improve the production process (3). This adaptive behavior improves the energy efficiency of enterprises at the micro level, while at the macro level, it shows the overall improvement of the intensity of environmental regulation. Relevant studies have confirmed that the strengthening of environmental regulation can promote the green transformation of enterprises (33).

#### 2.2.4 Green innovation drive

According to the innovation compensation theory, the clean heating policy provides dual incentives for green technology innovation by creating new market demand and exerting compliance pressure. This logic has also been verified in other policy situations. For example, the promotion of prefabricated buildings (11) and the application of new energy vehicles (14) have been proved to drive green technology progress through significant ‘innovation effect', thereby improving green energy efficiency (34). The implementation of the policy has driven the innovation investment in the research and development of clean heating technology and equipment manufacturing (35), and promoted the progress and diffusion of energy-saving and environmental protection technology (36). This innovation driven effect not only helps to reduce the cost of clean energy, but also improves energy efficiency through technological progress. Recent research shows that the inductive effect of environmental regulation on green technology innovation is increasing (11).

#### 2.2.5 The moderating role of regional capacity

The effectiveness of uniform command-and-control environmental regulation is endogenously conditioned by local endowments. We conceptualize this conditioning mechanism through the lens of “regional capacity”, a city' s composite ability to translate policy signals into green development outcomes. Recent resilience assessments across China' s major urban agglomerations (37) confirm that cities with higher initial resilience tend to exhibit stronger absorptive capacity, which directly supports our conceptualization of “regional capacity”. This capacity comprises four interconnected dimensions.

At the foundation lies scale-agglomeration capacity, proxied by city size and economic development level. Larger and more developed cities possess greater fiscal resources to co-finance subsidies, denser energy infrastructure to reduce transition costs, and more agglomerated talent and capital to support clean industry expansion, thereby strengthening policy resource mobilization and cost-sharing capability.

A second, structurally oriented dimension is structural transformation capacity, captured by initial GTFEE levels. Cities starting with higher efficiency face lower path dependence and can rapidly convert clean-heating-induced demand for equipment manufacturing and energy-saving services into local industrial upgrading. Conversely, cities with low initial efficiency may experience short-term “compliance cost” pressure, as fixed investments crowd out productive resources.

Equally critical is institutional implementation capacity, empirically represented by the government governance capability examined in the mechanism analysis. Clean heating policy, as a binding mandate embedded in official performance evaluation, depends critically on local governments' enforcement in environmental monitoring, cross-departmental coordination, targeted subsidy allocation, and post-installation operational support. Stronger institutional capacity reduces execution leakages and amplifies policy signals to micro-level actors.

Complementing these is knowledge absorption capacity, shaped by technological endowment, geographic location, and infrastructure connectivity. Cities well-endowed with high-tech industries can identify and internalize clean-heating-induced green technology demand through local innovation ecosystems, while high-speed rail hubs facilitate knowledge spillovers and resource reallocation across space. The role of geographic location, however, operates through a distinct logic specific to this policy context: non-coastal inland cities, where reliance on scattered coal heating is historically more entrenched, experience a larger marginal benefit from mandated fuel substitution. This direct abatement effect may outweigh the generic openness advantages typically associated with coastal regions, rendering inland cities the primary beneficiaries of the policy in terms of knowledge absorption and application.

These four dimensions are mutually reinforcing, jointly determining whether a city can convert the “compliance pressure” of environmental mandates into “innovation compensation” that raises GTFEE. This framework moves beyond the traditional “compliance cost vs. innovation compensation” dichotomy, offering a systematic account of the heterogeneous effects of environmental regulation.

## 3 Methodology and data sources

### 3.1 SBM-GML

To scientifically measure the green total factor energy efficiency (GTFEE), this study employs the non-radial, non-angular SBM directional distance with the global reference GML index (38), which can effectively solve the radial and angular measurement deviations the traditional DEA model, while fully considering the relaxation improvement and properly dealing with undesirable outputs (39). First, define the global production technology set PG that includes undesirable outputs, which incorporates all observed periods of time in the reference set to ensure intertemporal comparability. As shown in Equation 1:


$$\begin{array}{l} P^{G}=\{(x,y,b)|x\geq \sum_{t=1}^{T}\sum_{k=1}^{K}z_{k}^{t}x_{k}^{t},\;y\leq \sum_{t=1}^{T}\sum_{k=1}^{K}z_{k}^{t}y_{k}^{t},\;\\ \;b=\sum_{t=1}^{T}\sum_{k=1}^{K}z_{k}^{t}b_{k}^{t},\;\sum_{k=1}^{K}z_{k}^{t}=1,\;z_{k}^{t}\geq 0\} \end{array}$$ (1)

Among them, $\left(x_{k}^{t},y_{k}^{t},b_{k}^{t}\right)$ is the input, expected output and unexpected output vector of the kth decision-making unit in t period. $z_{k}^{t}$ is intensity variable, constraint condition $\overset{K}{\underset{k=1}{\sum }}z_{k}^{t}=1$ indicates that the production technology is variable return to scale (VRS).

For the evaluated DMU $\begin{array}{c} \left({\text{x}}_{\text{0}}^{t},{\text{y}}_{\text{0}}^{t},{\text{b}}_{\text{0}}^{t}\right) \end{array}$, its SBM distance function based on global directional distance is defined as shown in Equation 2:


$$\begin{array}{l} {\vec{S}}_{v}^{G}\left({\text{x}}_{\text{0}}^{t},{\text{y}}_{\text{0}}^{t},{\text{b}}_{\text{0}}^{t}\right)\text{=}max\frac{\frac{\text{1}}{M}\overset{M}{\underset{m\text{=1}}{\sum }}\frac{s_{m}^{x}}{x_{m\text{0}}^{t}}\text{+}\frac{\text{1}}{N\text{+}J}\left(\overset{N}{\underset{n\text{=1}}{\sum }}\frac{s_{n}^{y}}{y_{n\text{0}}^{t}}\text{+}\overset{J}{\underset{j\text{=1}}{\sum }}\frac{s_{j}^{b}}{b_{j\text{0}}^{t}}\right)}{\text{2}} \end{array}$$ (2)

Satisfy the constraints as shown in Equation 3:


$$\begin{array}{l} \begin{array}{cc} \overset{T}{\underset{t=1}{\sum }}\overset{K}{\underset{k=1}{\sum }}z_{k}^{t}x_{km}^{t}+s_{m}^{x}=x_{m0}^{t}, & m=1,...,M\\ \overset{T}{\underset{t=1}{\sum }}\overset{K}{\underset{k=1}{\sum }}z_{k}^{t}y_{kn}^{t}-s_{n}^{y}=y_{n0}^{t}, & n=1,...,N\\ \overset{T}{\underset{t=1}{\sum }}\overset{K}{\underset{k=1}{\sum }}z_{k}^{t}b_{kj}^{t}+s_{j}^{b}=b_{j0}^{t}, & j=1,...,J\\ s_{m}^{x}\geq 0,\;s_{n}^{y}\geq 0,\;s_{j}^{b}\geq 0,\;\overset{K}{\underset{k=1}{\sum }}z_{k}^{t}=1,\;z_{k}^{t}\geq 0 \end{array} \end{array}$$ (3)

In this model, the direction vector has been specifically set as $\text{g}\text{=}\left({\text{g}}^{x},{\text{g}}^{y},{\text{g}}^{b}\right)\text{=}\left({\text{x}}_{\text{0}}^{t},{\text{y}}_{\text{0}}^{t},-{\text{b}}_{\text{0}}^{t}\right)$, and has been directly substituted into the objective function. This setting is a standard practice for the SBM model to deal with undesired outputs, and its economic meaning is to the maximum possible expansion of the expected output and the maximum possible reduction of the undesired output under the existing level of inputs. (*s*^*x*^, *s*^*y*^, *s*^*b*^) are the slack for input excess, expected output insufficiency, and undesired output excess, respectively.

The GML productivity index from period t to t+1 is calculated using the above distance function, as shown in Equation (4):


$$\begin{array}{l} GML_{t}^{t\text{+1}}\text{=}\frac{\text{1+}{\vec{S}}_{v}^{G}\left({\text{x}}^{t},{\text{y}}^{t},{\text{b}}^{t}\right)}{\text{1+}{\vec{S}}_{v}^{G}\left({\text{x}}^{t\text{+1}},{\text{y}}^{t\text{+1}},{\text{b}}^{t\text{+1}}\right)} \end{array}$$ (4)

The GML index can be further decomposed into the product of the green total factor energy efficiency change ($GTFEEc{\text{h}}_{t}^{t\text{+1}}$) and the technological progress($GPTC_{t}^{t\text{+1}}$) as shown in Equation (5):


$$\begin{array}{l} GML_{t}^{t\text{+1}}\text{=}GTFEEch_{t}^{t\text{+1}}\times GPTC_{t}^{t\text{+1}} \end{array}$$ (5)

To carry out the regression analysis in the following, this study sets the value of the baseline (2006) green total factor energy efficiency level of city as 1, and uses the calculated period-by-period efficiency change index (GTFEEch) to multiply and construct the GTFEE level value for the DID that can be compared across periods.

### 3.2 Multi-period difference-in-differences model

To accurately identify the net effect of the northern clean heating policy, this study treats this phased policy implementation as a quasi-natural experiment, and builds the following-Multi-period Difference-in-Differences Model (21). As shown in Equation 6:


$$\begin{array}{l} GTFEE_{it}\text{=}\beta_{\text{0}}\text{+}\beta_{\text{1}}did_{it}\text{+}\lambda {\text{X}}_{it}\text{+}\mu_{i}\text{+}\gamma_{t}\text{+}\varepsilon_{it} \end{array}$$ (6)

Where *GTFEE*_*it*_ is the green total factor energy efficiency of city i in year t, which is the explained variable of this article. *did*_*it*_ is the core explanatory variable, a policy dummy variable (i.e., the DID term), which is composed of the interaction term of time dummy variable (time) and the dummy variable for the pilot cities (treated). If city i is included in the pilot scope of the clean heating policy in year t and beyond, *did*_*it*_ takes the value of 1, otherwise 0. **X**_*it*_ is a vector of city-level control variables that vary over time. μ_*i*_ and γ_*t*_ represent the city and time fixed effects, respectively, to control for unobserved factors that do not vary across individuals and vary with time in common. γ_*t*_ is a random error term. Coefficient β_1_ captures the average treatment effect of the policy implementation on the green total factor energy efficiency, which is the focus of this study.

### 3.3 Mechanism test model

In order to further explore the internal channel of clean heating policy affecting green total factor energy efficiency (GTFEE), we set up an impact mechanism test model based on the mediation effect framework (40, 41). The model is used to test whether the policy has an impact on GTFEE through four theoretical hypothesis paths, namely, industrial structure upgrading, government governance ability, environmental regulation intensity and green technology innovation. First, we assess the direct impact of the policy on the intermediary variable (M). As shown in Equation 7:


$$\begin{array}{l} M_{it}=\alpha_{0}+\alpha_{1}\times did_{it}+\lambda X_{it}+\mu_{i}+\gamma_{t}+\varepsilon_{it} \end{array}$$ (7)

Among them, *M*_*it*_ is a group of intermediary variables, representing respectively: industrial structure upgrading, government governance ability, environmental regulation intensity and green technology innovation. Coefficient α_1_ reflects the average treatment effect of the northern clean heating policy on the corresponding intermediary variables.

Secondly, in order to investigate the role of these intermediary variables in the process of policy affecting GTFEE, we introduced the interaction term between policy variables and intermediary variables into the benchmark model. As shown in Equation 8:


$$\begin{array}{l} GTFEE_{it}=\beta_{0}+\beta_{1}\times did_{it}+\beta_{2}\times M_{it}+\beta_{3}\times \left(did_{it}\times M_{it}\right)\\ \;+\lambda X_{it}+\mu_{i}+\gamma_{t}+\varepsilon_{it} \end{array}$$ (8)

The model aims to test whether the policy effect can be transmitted or strengthened through the intermediary variable M. Interactive term coefficient β_3_ is the key evidence to judge whether the variable is an effective transmission channel. If β_3_ significant, it indicates that in cities with different levels of intermediary variable M, the promotion effect of clean heating policy on GTFEE is different, which confirms the existence of this variable as an impact mechanism.

All models control the same set of variables at the city level as the benchmark regression *X*_*it*_, and include urban fixed effect μ_*i*_ and fixed effect with year γ_*t*_. The standard error is adjusted by clustering at the city level.

### 3.4 Variable and data

#### 3.4.1 Explained variable

The explained variable of this study is green total factor energy efficiency, which is measured by non-radial and non-angular SBM directional distance function and global GML index. The input indicators include: capital stock (calculated by using the perpetual inventory method, based on 2006, unit: 100 million yuan), labor force (number of employees per unit at the end of the year, 10,000 people) and total energy consumption (10,000 tons of standard coal). The expected output is the real GDP of the region (billion-yuan, flat reduction based on 2006). Unexpected output includes industrial sulfur dioxide emissions, industrial wastewater emissions and industrial smoke (powder) dust emissions (both in tons). This model can effectively deal with the unexpected output and avoid the measurement bias of the traditional DEA model. This comprehensive index can capture the synergy efficiency of economic output, energy consumption and environmental pollution, and is the core index to evaluate the performance of regional green transformation.

#### 3.4.2 Explanatory variable

The core explanatory variable is the multi-period difference-in-differences term constructed, that is, the interaction between the virtual variable of policy implementation time and the virtual variable of pilot cities. This variable is used to identify the net causal effect of clean heating policy on green total factor energy efficiency, and its construction method is in line with the recent frontier measurement standard for multi period policy evaluation (21, 42).

#### 3.4.3 Control variable

In order to control other potential confounding factors, this study introduces a series of urban level control variables, including population size, degree of opening up, degree of government intervention, industrial structure, education expenditure and urbanization level. The selection of these variables refers to the recent high-level research in the field of environmental economics (43) to ensure the preciseness and comparability of the model setting (44, 45).

#### 3.4.4 Mechanism variable

In order to test the intermediary path in the theoretical framework, this study sets up four types of mechanism variables: industrial structure upgrading, government governance ability, environmental regulation intensity and green technology innovation. These variables are measured by the value-added and proportion of the tertiary industry, the ratio of fiscal expenditure level to GDP, the pollution intensity of sulfur dioxide emissions per unit of GDP and the data of green patent applications, which have been widely used and verified in recent journal literature (4, 46). Variable definitions are shown in Table 1, and descriptive statistics of the variables are shown in Table 2.

**Table 1:** Variables definition.

Type	Symbol	Variable
Explained variable	*GTFEE*	Green total factor energy efficiency
Explanatory variable	*DID*	The interaction term of time dummy variable (time) and pilot cities (treated)
Control variable	*POP*	Population size
	*OPEN*	Degree of openness
	*GOV*	The extent of government intervention
	*IND*	Industrial structure
	*EDU*	Expenditure on education level
	*URBAN*	Urbanization level

**Table 2:** Variable descriptive statistics.

Variable	Min	Max	Mean	Std.Dev	Obs
*GTFEE*	0.021	1.177	0.321	0.131	5,112
*DID*	0	1	0.040	0.195	5,112
*POP*	2.888	8.143	5.868	0.697	5,112
*OPEN*	2.72	10.44	2.90	0.573	5,112
*GOV*	0.043	1.027	0.183	0.102	5,112
*IND*	50.11	99.97	86.94	8.470	5,112
*EDU*	0.020	0.792	0.180	0.044	5,112
*URBAN*	1.609	7.942	5.728	0.926	5,112

#### 3.4.5 Data source

The panel data used in this study cover the prefecture-level cities in northern China from 2006 to 2022. The input-output data, core explanatory variables, control variables and mechanism variables data required for the measurement of Green Total Factor Energy Efficiency (GTFEE) are mainly from the yearbooks of China' s Urban Statistics, China' s Energy Statistics and the statistical yearbooks of provinces and municipalities over the years. A small amount of missing data was supplemented using the exponential smoothing method. All the original data were based on publicly available statistical data to ensure the reproducibility scientificity of the study.

## 4 Analysis of empirical results

### 4.1 Benchmark regression results

In order to accurately identify the net effect of the northern clean heating policy on the GTFEE of the pilot cities, this study uses the stepwise regression method to include various control variables on the basis of controlling the fixed effect of cities and years. The results shown in Table 3 indicate that the estimation coefficient of the core explanatory variable did is significantly positive at the 1% level across all model specifications, demonstrating strong robustness. Specifically, the did coefficient in the basic model (1) without control variables is 0.090; With the gradual introduction of control variables, the coefficient value remained stable, and finally was calibrated as 0.065 in the complete model (7), which showed that the clean heating policy increased the GTFEE of pilot cities by 6.5% on average, and the implementation of the policy had a significant positive impact.

**Table 3:** Stepwise regression test results.

Variable	(1)	(2)	(3)	(4)	(5)	(6)
DID	0.090^***^	0.084^***^	0.080^***^	0.073^***^	0.065^***^	0.065^***^
	(0.022)	(0.021)	(0.020)	(0.020)	(0.020)	(0.020)
Pop		0.069^***^	0.059^***^	0.061^***^	0.059^***^	0.056^***^
		(0.011)	(0.011)	(0.011)	(0.010)	(0.015)
Open			−0.049^***^	−0.044^***^	−0.039^***^	−0.039^***^
			(0.014)	(0.014)	(0.014)	(0.015)
Gov				0.142^***^	0.113^***^	0.116^***^
				(0.039)	(0.043)	(0.044)
Ind					0.004^***^	0.004^***^
					(0.001)	(0.001)
Edu					−0.096	−0.095
					(0.086)	(0.087)
Urban						0.005
						(0.015)
Year FE	Yes	Yes	Yes	Yes	Yes	Yes
City FE	Yes	Yes	Yes	Yes	Yes	Yes
N	5,112	5,112	5,112	5,112	5,112	5,112
adj. R^2^	0.478	0.487	0.506	0.509	0.522	0.522

Standard errors in parentheses ^*^p < 0.1, ^**^p < 0.05, ^***^p < 0.01.

Through in-depth analysis of the control variables, we further revealed the multidimensional factors affecting GTFEE. Population size, government intervention and industrial structure upgrading all showed significant positive effects, which respectively confirmed the positive contributions of agglomeration economy, government regulation and industrial transformation to green development. However, the degree of opening to the outside world is significantly negative, suggesting that foreign capital introduced in some regions may still be concentrated in high-energy consumption industries. The impact of education investment and urbanization level has not passed the significance test, and its mechanism may be more complex or have time lag effect. These findings together show that in addition to the positive impact of clean heating policy, the improvement of urban green efficiency is also driven by a series of economic and social factors.

### 4.2 Robustness test

#### 4.2.1 Parallel trends test

**Figure 3:**
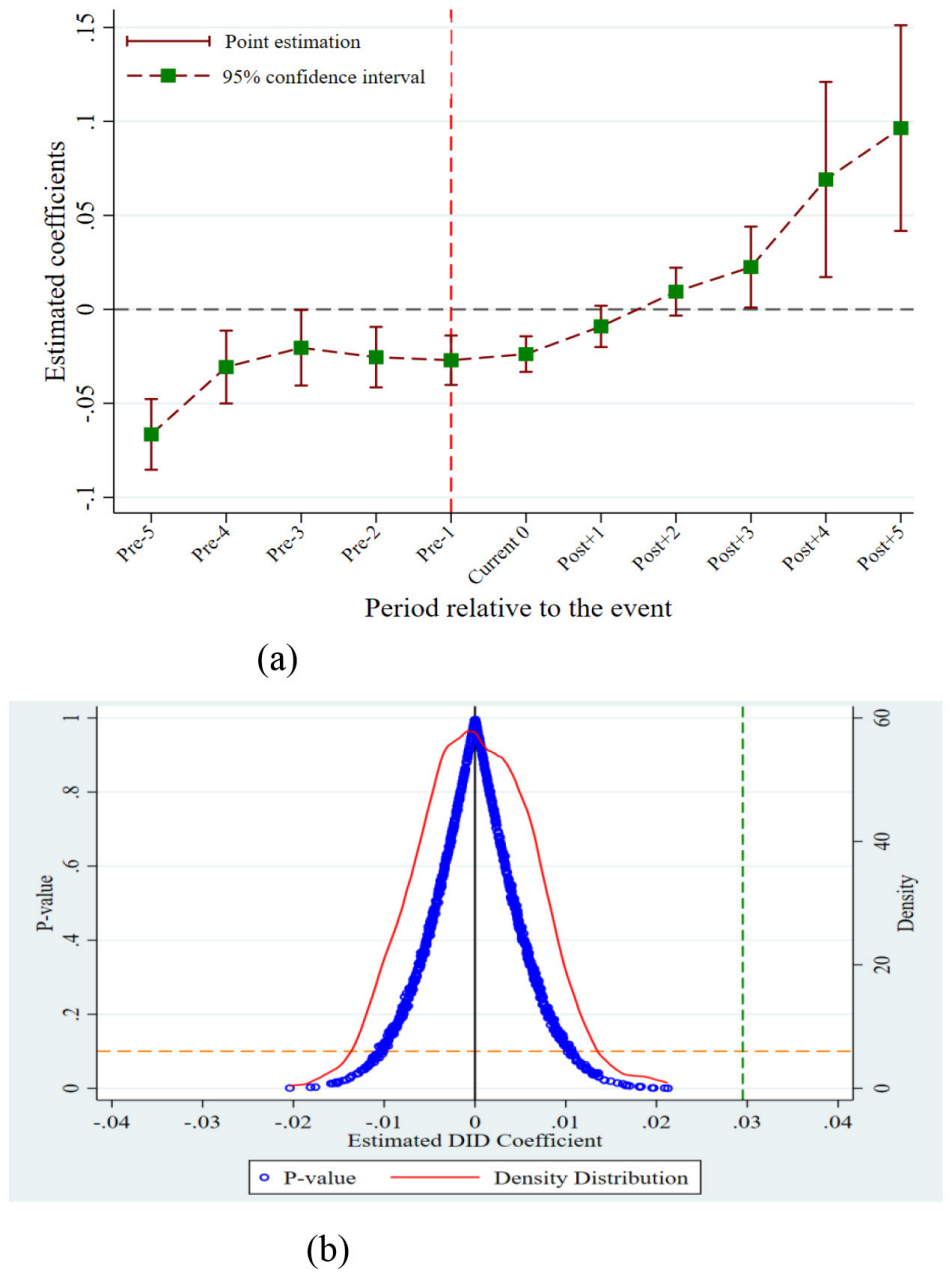
Parallel trends test and placebo test result chart. **(a)** Parallel trends test results; **(b)** Placebo test results.

The validity of the double-difference model relies on the parallel-trend assumption being met between the treated and control groups. The study traces the dynamic effect of policy by event study method (47) and the results are shown in Figure 3A. In the periods before the policy implementation, the estimated coefficients fluctuate around 0 and the confidence intervals contain 0, indicating that there is no significant trend between the treatment and control groups before the policy shock, thus satisfying the parallel trend assumption (48). After the implementation of the policy, the coefficient immediately turned significantly positive and continued thereafter, indicating that the clean heating policy has a significant and sustained improvement effect on the total factor energy efficiency (GTFEE).

#### 4.2.2 Placebo test

To rule out the possibility that unobserved factors drive the results, the placebo test was conducted in this study. The distribution of the virtual policy estimator was constructed by piloting the policy in randomly assigned cities and repeating the simulation 1,000 times. The results are shown in Figure 3B), where the kernel density of the simulated coefficients clusters closely around zero, while the true estimates from the benchmark regression are far in the right tail of this distribution. This result provides strong evidence that the policy effects observed in the benchmark regression are not due to a random factor.

#### 4.2.3 Change the time window

To test the sensitivity of the findings to the sample time range, this study changes the time window by dropping the policy implementation year and the following specific years for regression. As shown in Table 4, the coefficient of the core explanatory variable DID remains significantly positive under both the condition of excluding the data of 2017 and 2022 and the more stringent condition of further excluding the data of 2018 and 2021. This proves that the policy effect not suffer from excessive influence of a specific year or potential concurrent shocks, and the conclusion is robust.

**Table 4:** Change time window regression result table.

Variable	(1)	(2)	(3)	(4)
DID	0.093^***^	0.040^*^	0.088^***^	0.059^**^
	(0.021)	(0.021)	(0.023)	(0.023)
_cons	0.312^***^	−0.565	0.305^***^	0.276^***^
	(0.001)	(0.589)	(0.001)	(0.023)
Control variable	No	Yes	No	Yes
Year FE	Yes	Yes	Yes	Yes
N	4,170	4,170	3,614	3,614
adj. R^2^	0.565	0.670	0.588	0.641

(1, 2) To remove the data results of 2017 and 2022.

(3, 4) are the data results removing the data of 2017, 2018, 2021, and 2022.

Standard errors in parentheses ^*^p < 0.1, ^**^p < 0.05, ^***^p < 0.01.

#### 4.2.4 Tailoring treatment

Considering that extreme values may interfere with the estimation results, this study winsorized the main continuous variables at the 1% level. The regression results shown Table 5 indicate that the DID coefficient is still significant.

**Table 5:** Regression results of tailing treatment.

Variable	(1)	(2)
DID	0.028^***^	0.029^***^
	(0.007)	(0.007)
_cons	0.221^***^	−0.021
	(0.004)	(0.164)
Control variable	No	Yes
Year FE	Yes	Yes
N	4,726	4,726
adj. R^2^	0.296	0.305

Standard errors in parentheses ^*^p < 0.1, ^**^p < 0.05, ^***^p < 0.01.

#### 4.2.5 Instrumental variable method

To alleviate the potential endogeneity problem (e.g., reverse causality) in the implementation of the policy, this study introduces historical geographical variables as instrumental to conduct a two-stage least squares (2SLS) estimation. Table 6 reports the regression results using different combinations of instrumental variable, i.e., “average slope”, “average slope and river density” etc. Models from 1 to 5 are DID = average slope; DID = average slope river density; DID = average slope exchange rate; DID = average slope river density exchange; and DID = average slope river density exchange rate post office number. The coefficient of DID is significantly positive at the 1% level under all settings, and the first-stage F-statistic is much greater than the value under the 10% error level, which effectively rejects the hypothesis of weak instrumental variable. This confirms that the promotion effect of clean heating policy on GTFEE is still and reliable after considering endogeneity.

**Table 6:** Tool variable inspection results.

Variable	(1)	(2)	(3)	(4)	(5)
DID	0.383^***^	0.600^***^	0.279^***^	0.329^***^	0.184^***^
	(0.103)	(0.123)	(0.102)	(0.096)	(0.059)
Control variable	Yes	Yes	Yes	Yes	Yes
Year FE	Yes	Yes	Yes	Yes	Yes
N	5,112	5,112	5,112	5,112	5,112
adj. R^2^	0.010	−0.622	0.172	−0.057	0.151

Standard errors in parentheses ^*^p < 0.1, ^**^p < 0.05, ^***^p < 0.01.

#### 4.2.6 Switch matching method

In order to mitigate the sample selection bias caused by non-random assignment of policy pilot cities, we use the propensity score matching double difference (PSM-DID) method to test the robustness. We selected the level of economic development, industrial structure, the degree of government intervention, education expenditure and urbanization level as covariates, and used the kernel matching method to construct a comparable control group for the treatment group.

**Figure 4:**
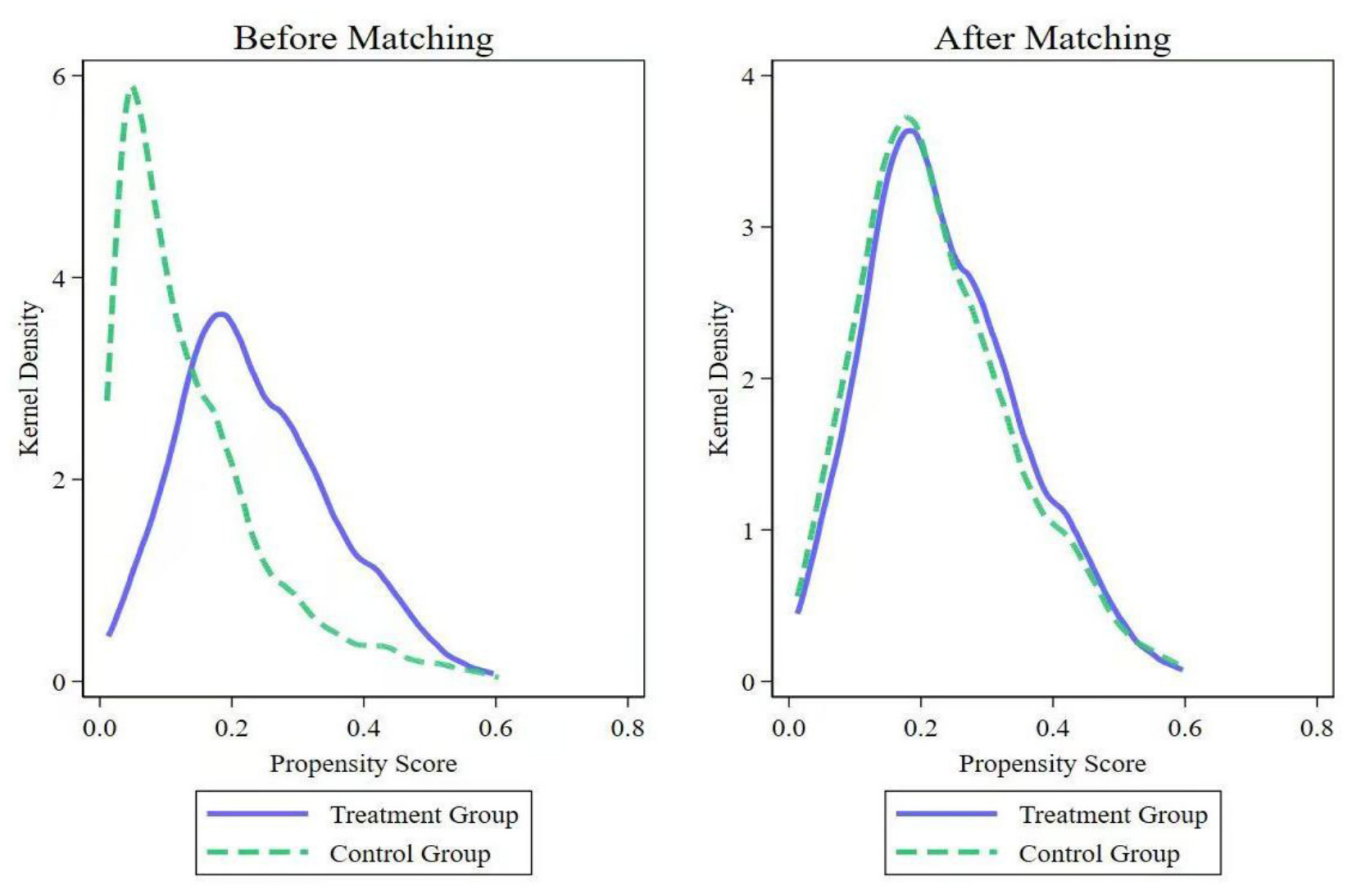
Comparison diagram of kernel density function.

The matching quality test results show that the matching effect is good. The standardized deviation chart shows that the absolute value of standardized deviation of all covariates after matching is significantly reduced. The comparison chart of kernel density function shows that the distribution of propensity scores in the matched post-processing group and the control group tends to be consistent, meeting the balance assumption (as shown in Figure 4).

Table 7 reports the estimated results of PSM-DID. Columns (1) and (2) are the benchmark regression results of the original sample, and columns (3) and (4) are the estimated results of the matched sample. The results show that under the same setting of control variables, the did coefficients in the matched samples are close to the original samples, and both remain significant at the 1% level. Specifically, in the complete model containing all control variables, the did coefficient of the matched sample is 0.031, which is slightly different from the coefficient of the original sample of 0.030. This result shows that the promotion effect of clean heating policy on green total factor energy efficiency is still robust after controlling the sample selectivity bias.

**Table 7:** PSM-DID regression results.

Variable	(1)	(2)	(3)	(4)
DID	0.027^***^	0.030^***^	0.027^***^	0.031^***^
	(0.007)	(0.007)	(0.007)	(0.007)
_cons	0.317^***^	−0.160	0.317^***^	−0.150
	(0.001)	(0.187)	(0.001)	(0.187)
Control variable	No	Yes	No	Yes
Year FE	Yes	Yes	Yes	Yes
N	5,112	5,112	5,112	5,112
adj. R^2^	0.689	0.696	0.690	0.697

Standard errors in parentheses ^*^p < 0.1, ^**^p < 0.05, ^***^p < 0.01.

#### 4.2.7 Eliminate other policy interference

To disentangle the core policy effect from potential confounding arising from other concurrent environmental policies during the observation period, this study further accounts for two major contemporaneous environmental policies: the low-carbon city pilot policy and the carbon emission trading pilot policy. Specifically, based on the pilot timing and pilot cities of each policy, corresponding DID dummy variables are constructed and incorporated into the baseline regression model. The regression results, as shown in Table 8, indicate that in Model (1), which controls for the low-carbon city pilot policy, the coefficient of the core explanatory variable DID is 0.061, significant at the 1% level; in Model (3), which controls for the carbon emission trading pilot policy, the coefficient of the core explanatory variable DID is 0.064, also significant at the 1% level. Compared with the baseline regression results, both the magnitude and significance of the coefficient on the core explanatory variable remain largely unchanged. Moreover, the low-carbon city pilot policy variable itself exhibits a significantly positive effect in Model (1). These findings suggest that the core conclusion of this study is not driven by concurrent environmental policies, further validating the robustness of the baseline regression results.

**Table 8:** Eliminate other policy interference.

Variable	(1) Low- carbon city pilot	(2) Carbon emissions trading pilot
DID	0.061^***^	0.064^***^
	(0.019)	(0.020)
_cons	−1.282^**^	−1.710^***^
	(0.578)	(0.593)
Control variable	Yes	yes
City FE	Yes	yes
N	4,777	5,058
adj. R^2^	0.607	0.600

Standard errors in parentheses ^*^p < 0.1, ^**^p < 0.05, ^***^p < 0.01.

### 4.3 Heterogeneity analysis

Based on the regional capacity framework, we examine policy heterogeneity along three groups of city characteristics, mapped onto the four capacity dimensions as follows. City size and economic development level operationalize scale-agglomeration capacity. Initial GTFEE captures structural transformation capacity. Geographic location and high-speed rail connectivity jointly reflect knowledge absorption capacity.Institutional implementation capacity, while not examined as a separate heterogeneity cut, is substantively represented by the government governance channel in the mechanism analysis (Table 9). Together, the heterogeneity and mechanism analyses constitute a progressive empirical test of the proposition that regional capacity endogenously shapes policy effectiveness.

**Table 9:** Mechanism inspection result table.

Variable	Industry-upgrade	Super-sbm	Gov-capacity	Super-sbm	Env-regulation	Super-sbm	Green-innovation	Super-sbm
DID	0.006^**^		0.222^***^		−0.096^***^		0.530^***^	
	(0.002)		(0.008)		(0.028)		(0.028)	
IND		0.006^***^						
		(0.002)						
GOV				0.067^***^				
				(0.015)				
ENV						−0.671^***^		
						(0.164)		
GRE								0.046^***^
								(0.009)
_cons	−0.294	−0.494^***^	4.671^***^	−0.794^***^	2.787^**^	−0.432^***^	−13.88^***^	−0.367^***^
	(0.258)	(0.098)	(0.435)	(0.099)	(1.049)	(0.073)	(0.595)	(0.051)
Control variable	Yes	Yes	Yes	Yes	Yes	Yes	Yes	Yes
Year FE	Yes	Yes	Yes	Yes	Yes	Yes	Yes	Yes
N	5,112	5,112	5,112	5,112	5,112	5,112	5,112	5,112
adj. R^2^	0.999	0.140	0.437	0.231	0.067	0.193	0.492	0.193

Standard errors in parentheses ^*^p < 0.1, ^**^p < 0.05, ^***^p < 0.01.

#### 4.3.1 Urban size heterogeneity

As shown in Table 10, the policy effect varies by city size. The policy effects of mega-cities and large cities are significantly positive. This indicates that mega-cities and large cities in the rapid development stage are most active in their policy response. In comparison, the policy effects of both mega-cities and small cities are insignificant, which may stem from the diminishing marginal returns of efficiency improvement faced by the former and the limited fiscal and technical capabilities of the latter. Midsize cities show the largest positive effect and are significant at the 1% level, highlighting their potential as targets for policy implementation.

**Table 10:** Test results of heterogeneity of urban development scale.

Variable	Megacity	Metropolis	Big city	Secondary city	Small city
DID	−0.017	0.074^**^	0.019^***^	0.086^***^	−0.011
	(0.036)	(0.029)	(0.007)	(0.029)	(0.025)
Control variable	Yes	Yes	Yes	Yes	Yes
Year FE	Yes	Yes	Yes	Yes	Yes
N	170	476	3,684	714	68
adj. R^2^	0.668	0.410	0.225	0.144	0.531

Standard errors in parentheses ^*^p < 0.1, ^**^p < 0.05, ^***^p < 0.01.

#### 4.3.2 Initial energy efficiency heterogeneity

For cities with a higher initial GTFEE, the policy generated a significant uplifting effect. As shown in Table 11, for cities with moderate initial efficiency, the policy effect, though small, is still significant. However, for the initial low-efficiency cities, the policy impact is significantly negative, revealing a “follow-the-cost” effect: the initial green transformation' s fixed investment poses a short-term burden for cities with weak foundations, potentially crowding out productive resources leading to a short-term decrease in their green total factor energy efficiency.

**Table 11:** Test results of scale heterogeneity of green energy efficiency.

Variable	Low efficiency	Mid efficiency	High efficiency
DID	−0.020^***^	0.018^***^	0.093^***^
	(0.004)	(0.004)	(0.020)
_cons	0.306^***^	0.320^***^	−1.502^***^
	(0.094)	(0.098)	(0.509)
Control variable	Yes	Yes	Yes
Year FE	Yes	Yes	Yes
N	1,704	1,704	1,704
adj. R^2^	0.356	0.106	0.078

Standard errors in parentheses ^*^p < 0.1, ^**^p < 0.05, ^***^p < 0.01.

#### 4.3.3 Geographical location, economic development and heterogeneity of high-speed rail infrastructure

The effect of policy is affected by geographical location, economic development level and infrastructure conditions. The heterogeneity test results of the three are shown in Table 12. The analysis shows that non-coastal inland cities, economically developed cities and high-speed rail hub cities enjoy more significant policy dividends. This highlights that the effectiveness of the clean heating policy not only depends on the local financial resources and economic base to bear the transformation costs, but also benefits from the stronger marginal effect of loose coal substitution in inland areas, as well as the knowledge and technology spillover and resource allocation optimization brought about by the high-speed rail network.

**Table 12:** Geographical location, economic development scale and heterogeneity of high-speed rail infrastructure.

Variable	HSR	NHSR	Coastal	Non-coast	Low level	High level
DID	0.103^***^	0.005	−0.022	0.042^***^	0.011	0.031^***^
	(0.014)	(0.007)	(0.027)	(0.007)	(0.013)	(0.010)
-cons	0.373	0.538^***^	−2.160^***^	0.697^***^	1.465^***^	−1.776^***^
	(0.323)	(0.206)	(0.467)	(0.202)	(0.240)	(0.276)
Control variable	Yes	Yes	Yes	Yes	Yes	Yes
Year FE	Yes	Yes	Yes	Yes	Yes	Yes
N	1,853	3,259	1,219	3,893	2,227	2,885
adj. R^2^	0.370	0.188	0.327	0.238	0.187	0.192

Standard errors in parentheses ^*^p < 0.1, ^**^p < 0.05, ^***^p < 0.01.

### 4.4 Mechanism inspection

In order to verify the four mediation paths proposed in the theoretical analysis, we conducted a systematic mechanism test. Before the empirical test, we clarify the statistical and conceptual independence between green technology innovation (measured by green patent applications) and green total factor energy efficiency (GTFEE, calculated by the SBM-GML index). Green patent applications capture urban green technology creation and innovation efforts, while GTFEE reflects the joint efficiency of energy, economic, and environmental systems integrating inputs, desirable outputs, and undesirable outputs. The two indicators follow distinct measurement logics, contain no overlapping variables or direct mathematical correlation, and satisfy the independence assumption required for mediation effect testing. This ensures that green technology progress is reliably identified as a causal transmission channel rather than a mechanical correlate of GTFEE. The mechanism test results are shown in Table 9, which clearly shows that the clean heating policy has jointly promoted the improvement of green total factor energy efficiency (GTFEE) through the four parallel and interrelated paths of industrial structure upgrading, government governance capacity strengthening, environmental regulation strengthening and green technology innovation. These paths do not exist in isolation, but are intertwined and synergetic in practice, which together constitute the multiple mechanisms of policy affecting regional energy efficiency.

Firstly, in terms of upgrading the industrial structure, the implementation of the policy has significantly accelerated the process of upgrading and greening the regional industrial structure. The clean heating policy directly impacts the traditional industries related to traditional coal mining, transportation and inefficient coal-fired equipment manufacturing through the mandatory replacement of loose coal. At the same time, it creates a huge and determined market demand for emerging clean industries such as natural gas wall mounted boilers, efficient electric heating equipment, energy-saving building materials and related installation, operation and maintenance services. This “creative destruction” process drives the transfer of capital, labor and other production factors from high energy consumption and high pollution sectors to technology intensive, clean and efficient sectors. The empirical results in Table 9 show that policies play a significant role in promoting industrial upgrading, and industrial upgrading variables themselves have a robust positive relationship with GTFEE. This shows that the policy effectively improves the resource and energy allocation efficiency of the entire economic system by guiding the transformation of economic structure to service and high-tech, which is an important structural channel to achieve the improvement of green all factor energy efficiency (49).

Secondly, the policy has effectively strengthened the governance capacity of local governments. As a political task that has been incorporated into the performance appraisal system of local officials and has strong constraints, the clean heating policy has greatly mobilized the implementation will and administrative initiative of local governments. In order to ensure the effective implementation of the policy, local governments must strengthen environmental monitoring and law enforcement, optimize the allocation and regulatory efficiency of financial subsidies, and actively coordinate multi stakeholders such as gas companies, power grid enterprises, equipment suppliers and residential users. This complex implementation process itself is a systematic strengthening and practical exercise of the environmental governance capacity of local governments. Stronger governance means that public resources can be more accurately and efficiently allocated to green and low-carbon fields. The results in Table 9 further confirm that the improvement effect of policies on government governance capacity is very significant, and there is a significant positive interaction effect between the enhanced governance capacity and GTFEE. This shows that the strengthening of governance capacity is not only the guarantee of policy implementation, but also the key enabling factor to amplify the final environmental and economic benefits of the policy.

Thirdly, the strengthening of environmental regulation and the “reverse forcing” mechanism still play an important driving role in the system, although its direct effect shows the characteristics of marginal decline in value. As a highly visible and mandatory environmental regulation signal, the clean heating policy clearly conveyed the central government' s firm determination to promote clean energy transformation to all micro entities in the market, thus profoundly changing the expectations of enterprises for the future policy environment and compliance costs. In order to cope with the current and expected tightening environmental protection requirements, enterprises not only need to passively meet the current clean heating equipment standards, but also may be “forced” to carry out forward-looking green technology research and development, process optimization and environmental management innovation, so as to avoid future compliance risks and potential costs. Table 9 shows that the direct effect of policy on the intensity of environmental regulation is negative, but the interaction between environmental regulation and GTFEE is significantly negative. This confirms the existence of the “forcing” logic: the strengthening of regulation in the short term will increase the cost burden of enterprises, but it is this pressure that forces enterprises to seek more fundamental and more efficient technology and management solutions, thus indirectly and effectively promoting the improvement of long-term energy efficiency in the dynamic process (50).

Finally, the core path is to stimulate green technology innovation by policies. According to the core assertion of “Porter Hypothesis”, properly designed environmental regulations can stimulate the compensation effect of innovation, so as to partially or completely offset the compliance cost. The clean heating policy has spawned a clean energy technology and equipment market with considerable scale and rapid growth, providing a clear market demand pull and profit incentive for green technology innovation in related fields. At the same time, the mandatory compliance pressure brought by the policy also constitutes a strong technical driving force. Under the joint action of these two forces, the RandD investment and innovation activities of enterprises are guided to green technology fields such as energy-saving technology, clean energy utilization and intelligent control system. The results in Table 9 strongly support this mechanism: policies have played an extremely significant role in promoting green innovation activities, and green innovation has been proved to be a key direct channel to enhance GTFEE. These innovations are the ultimate driving force to improve total factor energy efficiency (51), because they directly improve the technical efficiency of energy from input to expected output.

The mechanism test confirmed that the northern clean heating policy is not through a single dimension, but through the four parallel intermediary channels of reshaping the regional economic structure, governance mode, regulatory environment and innovation system, which work together to improve the GTFEE, forming a multi-dimensional and systematic policy impact framework.

## 5 Discussion

The empirical analysis results of this study show that the northern clean heating policy has effectively improved the green total factor energy efficiency of pilot cities, but this effect has significant heterogeneity and is regulated by the comprehensive threshold of “regional capacity” (52). Specifically, in cities with diversified industrial base, strong technological endowment, outstanding administrative efficiency, high economic level and perfect infrastructure, the green efficiency gain generated by the policy is more significant. This finding reveals that the final effect of environmental regulation depends not only on the design of the policy itself, but also on the economic structure, innovation ecology and governance system of the place where the policy is implemented (53).

The dialogue between this study and cutting-edge literature deepens our understanding of the effect of mandatory environmental regulation. Firstly, this paper provides new evidence for the applicability of the “Porter Hypothesis” in the context of mandatory policies from the urban level in China, which is consistent with the conclusion of recent studies that strict environmental regulation can stimulate innovation and enhance long-term competitiveness (54). This finding also resonates with recent work on urban ecological resilience, where polycentric spatial structures are shown to either buffer or amplify environmental policy impacts (55). At the mechanism level, this paper also verifies the three key paths of industrial structure upgrading, government governance strengthening and green technology innovation, which supports the view that the effectiveness of environmental policy is achieved through the coordinated evolution of economic structure, institutional environment and innovation system (36). The core marginal contribution of this study is to systematically identify and integrate the multidimensional composition of “regional capabilities”. Although previous studies have discussed the independent role of resource dependence (60), economic development (46) or transportation facilities (61), this study reveals how these elements together constitute a region' s comprehensive ability to transform policy signals into green development outcomes through a unified framework (56). This provides a more complete theoretical explanation for the huge regional differences in the effects of environmental policies (23, 47).

Simultaneously, several limitations should be noted. First, constrained by prefecture-level data, the proxies for green innovation and government governance are relatively coarse and may not capture the full nuance of micro-level behavioral responses. Second, the policy variable is constructed based on the timing of official pilot designations, while the actual intensity of local enforcement may differ across cities, potentially attenuating the precision of the treatment estimate.

These limitations point to meaningful directions for future work. At the micro level, firm-level energy and patent quality data could be employed to more precisely trace how the policy reshapes enterprises' investment and innovation decisions. At the spatial level, spatial econometric or gravity-type models could be applied to test for technology spillovers or carbon leakage effects across metropolitan areas and urban agglomerations (57). Extending the evaluation framework to social welfare outcomes, such as energy poverty alleviation, public health, and employment structure, would capture a broader spectrum of policy benefits. Methodologically, machine-learning-based heterogeneity analysis could help systematically identify and rank the regional conditioning factors, enabling more precise targeting of future environmental instruments. In general, this study offers an integrated perspective for understanding the heterogeneous effects of mandatory environmental regulation under different regional conditions and provides key evidence for advancing governance from “one size fits all” to “precise implementation”. Addressing the above limitations in future research is expected to yield more systematic insights at both theoretical and policy levels.

## 6 Conclusion

The empirical results of this study show that the deep value of clean heating policy, as an imperative environmental regulation, is that it has successfully catalyzed the systematic transformation of the development mode of pilot cities and driven the overall leap of the GTFEE. This proves that properly designed mandatory policy tools can go beyond end of pipe governance and become an effective lever to promote the green, high-quality and qualitative transformation of the economic system.

At the theoretical level, the breakthrough of this study lies in the construction of an integrated analytical framework of “regional capacity”, thus breaking the traditional binary opposition perspective. The framework clarifies that the size of policy dividends is essentially rooted in the inherent endowment and absorptive capacity of local economic and social systems. Accordingly, future environmental governance can be committed to precision and contextualization, and future policy design can be committed to activating the synergy between technological innovation, structural upgrading and governance optimization, so as to transform environmental constraints into development opportunities under specific regional conditions. The conclusion of this study can provide theoretical support and empirical basis for promoting the precise and differentiated environmental governance model.

## Data Availability

The original contributions presented in the study are included in the article, further inquiries can be directed to the corresponding authors.
